# Ancestry inference using reference labeled clusters of haplotypes

**DOI:** 10.1186/s12859-021-04350-x

**Published:** 2021-09-25

**Authors:** Yong Wang, Shiya Song, Joshua G. Schraiber, Alisa Sedghifar, Jake K. Byrnes, David A. Turissini, Eurie L. Hong, Catherine A. Ball, Keith Noto

**Affiliations:** AncestryDNA, San Francisco, CA 94107 USA

**Keywords:** ARCHes, Ancestry inference, Haplotype modeling, Local ancestry, HMM, RFMix

## Abstract

**Background:**

We present ARCHes, a fast and accurate haplotype-based approach for inferring an individual’s ancestry composition. Our approach works by modeling haplotype diversity from a large, admixed cohort of hundreds of thousands, then annotating those models with population information from reference panels of known ancestry.

**Results:**

The running time of ARCHes does not depend on the size of a reference panel because training and testing are separate processes, and the inferred population-annotated haplotype models can be written to disk and reused to label large test sets in parallel (in our experiments, it averages less than one minute to assign ancestry from 32 populations using 10 CPU). We test ARCHes on public data from the 1000 Genomes Project and the Human Genome Diversity Project (HGDP) as well as simulated examples of known admixture.

**Conclusions:**

Our results demonstrate that ARCHes outperforms RFMix at correctly assigning both global and local ancestry at finer population scales regardless of the amount of population admixture.

**Supplementary Information:**

The online version contains supplementary material available at 10.1186/s12859-021-04350-x.

## Background

Admixture has played an important role in shaping patterns of genetic variation among humans and other species. It is of interest at both population and individual levels and has motivated a large body of research into population demography [1, 2] and population stratification [3] in association studies. It has also fueled public interest in direct-to-consumer services that provide estimates of ancestry proportions. In such applications, a consumer typically submits a DNA sample through a saliva collection kit and receives an individual-level report of their ancestral make-up based on genotype data.

Over the past decade, many tools have been developed to infer individual-level ancestry. One set of methods only infers global ancestry proportions, some of which model the probability of the observed genotypes using ancestry proportions and population allele frequency [4], while others use cluster analysis and principal component analysis [5]. Another set of methods infer ancestral origin for genomic segments, which are then averaged over the entire genome. These methods use either SNPs (Single Nucleotide Polymorphisms) or a sequence of SNPs (*i.e.* haplotypes) as the observed variables, and estimate ancestry in each segment of the genome (called local ancestry). Compared to SNPs, haplotypes contain richer information, and can be especially powerful in differentiating geographically close populations [6]. Among existing haplotype-based methods, both Chromopainter [6] and HAPMIX [7] use the Li and Stephen’s haplotype copying model [8], whereas RFMix [9] uses a random forest approach, training classifiers on haplotype features in a reference panel and using a linear-chain conditional random field to model the conditional distribution of local ancestry given observed haplotypes.

As the size of public and private genotype datasets grows (*e.g.,* Ancestry has processed over 15 million human genomes), there is an increased need for methods that can efficiently and accurately perform ancestry inference on a large number of samples. Here we describe ARCHes (**A**ncestry inference using **R**eference labeled **C**lusters of **H**aplotyp**es**), a method that leverages reference panel labeled haplotype models to estimate diploid ancestry locally throughout the genome. ARCHes first uses a large cohort of unlabeled haplotypes to create BEAGLE haplotype-cluster models [10], which are efficient at phasing and measuring haplotype frequency, for each of a number of local “windows” across the genome. The haplotype clusters of the BEAGLE models are then annotated with the probability that haplotypes from various populations belong to those clusters. For a given test individual, ARCHes computes a probability distribution over the possible population assignments for the test individual’s two haplotypes, and uses a genome-wide hidden Markov model to assign diploid ancestry.

Previous studies have shown that RFMix [9] outperforms ADMIXTURE [4] in both global and local ancestry estimation [11]. RFMix generally performs well at assigning ancestry at continental level but, we will demonstrate, can struggle at regional level assignment, where populations may not be very differentiated. ARCHes is capable of differentiating nearby populations and performing recent ancestry inference (e.g., 1–12 generations ago) at a much finer scale.

## Results

### A summary of our approach

Our approach can be divided into two major phases, training and testing. The training phase consists of (1) building BEAGLE [10] haplotype models from a large cohort of phased data that do not have population labels, and (2) “annotating” those models with population-haplotype information from a separate population reference panel consisting of unphased examples each labeled with a population. We build a haplotype model for each 1001 windows that collectively cover the entire autosome (each window is about 75 SNPs and 3.5 cM). These models are built from phased data, which can be phased with BEAGLE or other phasing software. In our experiments, we build them from a cohort of 50,000 individuals (100,000 haplotypes) phased with Eagle [14]. The haplotype models are directed acyclic graphs with nodes that represent clusters of similar haplotypes and have probabilistic transitions between nodes. Next in the training phase, we record how likely the genotypes of an unphased reference panel are to belong to each of these haplotype clusters. We refer to this process as annotating the haplotype models—it gives us a probability for each node in the haplotype models and each population in our reference panel, that a haplotype from that population belongs to the haplotype cluster the node represents. Once the training phase is complete, we use only the annotated haplotype models which are fixed throughout the testing phase. The testing phase involves computing the likelihood that a test genotype has a haplotype belonging to any haplotype cluster (node in the models) and, using the population annotations for the cluster, computing the likelihood that the two haplotypes of the test genotype are explained by any pair of populations. We then use a genome-wide hidden Markov model (HMM) to model the changes in population assignment across the genome that best explain the test genotype.

The details of this approach are given in the Methods section below. We emphasize that the training phase need only be carried out once, and that the testing phase can then be applied to efficiently classify an arbitrary number of test genotype examples. If we obtain new population reference panel examples, we can use them to supplement the model annotations, even introducing new populations, but we must retrain completely to change the unlabeled phased cohort or window definitions.

### Accuracy for single-origin individuals

**Fig. 1:**
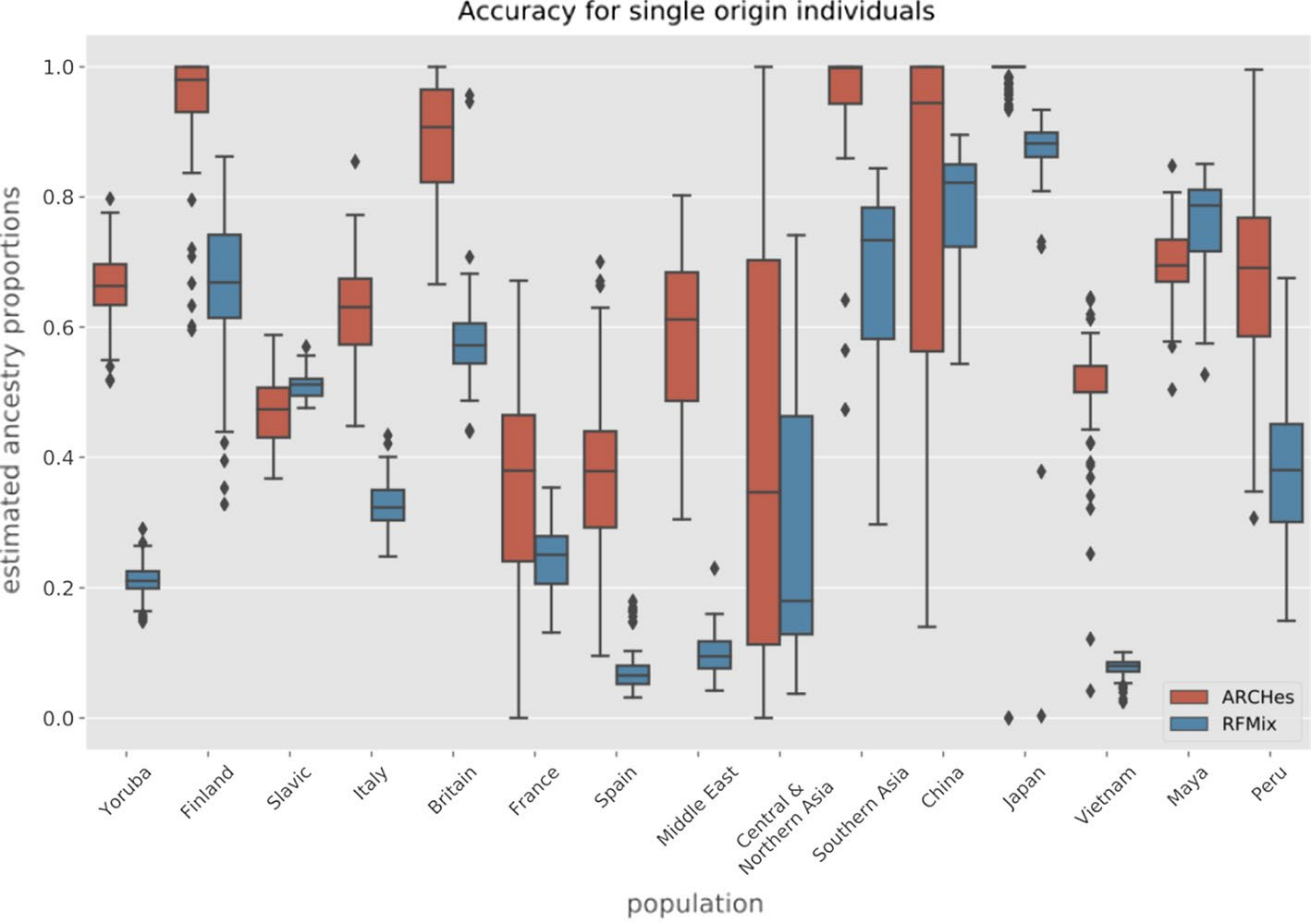
Boxplot of the estimated ancestry proportions for single-origin individuals from each testing population comparing ARCHes and RFMix

We built our reference panel using genotypes from proprietary data representing 32 population regions. We then applied ARCHes on individuals from 1000 genomes [12] and HGDP [13], representing 15 regions. (Lists of populations and associated sample sizes for both training and testing data are in Additional file 1: Tables S1 and S2, and we describe all experimental methodology in detail, including the parameter settings for both ARCHes and RFMix in the Methods section below.) ARCHes predicts on average 66.1% of the ancestry in this test set to be from the correct region (Fig. 1). The rest of the ancestry mainly came from nearby regions (Additional file 1: Fig. S2). ARCHes performs well at separating different countries within Africa, Europe, and Asia. In comparison, RFMix predicts on average 43.5% of the ancestry to be from the correct region, and the rest of the ancestry mainly came from neighboring regions, suggesting that RFMix is accurate for continental level assignments but performs less well at finer scales (Table 1).

**Table 1:** The performance of ARCHes and RFMix on various test sets

Test set group	Test set	Global concordance, average over test sets (proportion of test sets with superior performance)		Local concordance, average over test sets (proportion of test sets with superior performance)	
		ARCHes	RFMix	ARCHes	RFMix
1000 Genomes and HGDP	Single-Origin Testset Examples	**66.1% (13/15)**	43.5% (2/15)	**66.1% (13/15)**	43.5% (2/15)
Simulated admixture	45% Native American, 50% European, 5% African	**72.3% (1/1)**	65.7% (0/1)	**47.8% (1/1)**	18.5% (0/1)
Simulated admixture from 16 Pairs of neighboring regions	50%-50% admixed (2 simulation founders)	**60.1% (11/16)**	48.9% (5/16)	**58.8% (14/16)**	41.8% (2/16)
	Approx. 25%-75% Admixed (4)	**63.6% (13/16)**	51.8% (3/16)	**60.1% (14/16)**	44.7% (2/16)
	Approx. 12.5%-87.5% Admixed (8)	**65.2% (13/16)**	51.2% (3/16)	**62.6% (14/16)**	46.0% (2/16)
	Approx. 6.25%-93.75% Admixed (16)	**66.2% (14/16)**	50.0% (2/16)	**64.5% (14/16)**	47.0% (2/16)

Global concordance is the intersection between the estimated amounts for each region and the amount present in a test example, and local concordance is the number of correct assignments to each genomic window. For single-origin test examples, these measures are the same

### Accuracy for simulated admixed individuals

In order to evaluate the global and local accuracy on admixed individuals, we need to know the correct ancestry throughout the genome, so we manufactured test examples from the 1000 Genomes and HGDP data. We simulated 100 individuals using forward simulation with a pedigree mimicking Latino population history in which founders admixed 12 generations ago with 45% Native American, 50% European and 5% African ancestry. This dataset tests ARCHes’s power to differentiate continental level admixture as well as its ability to differentiate the subregions that an individual’s continental ancestry comes from.

To evaluate overall global performance on these test sets, we compute concordance as the size of the intersection between true and estimated proportions, which is the sum, for each population, of the smaller of the true global proportion and the estimated global proportion. We measured local accuracy as the proportion of genomic windows with correct diploid population assignments regardless of phase, with half credit given to a window assignment that has one population correct but the other incorrect. We find that ARCHes accurately recovers both global ancestry assignments and diploid local ancestry assignments, with average concordances of 72.3% and 47.8%, respectively (Additional file 1: Fig. S4). RFMix achieves 65.7% global ancestry concordance but failed to infer the local assignments correctly, with average diploid local ancestry concordance of 18.5%. This is due to difficulties that RFMix has in differentiatiating subregions within Europe and between Maya and Peru. The continental-level global and local concordance is 89.1% and 64.1% respectively for ARCHes, and 73.1% and 34.2% respectively for RFMix.

### Distinguishing sub-continental regions

Next, we simulated genotypes for individuals with ancestry from 16 pairs of neighboring regions to test each approach’s ability to distinguish between them at global and local genomic scales. Specifically, we construct test examples that are 1/2, 1/4, 1/8, or 1/16 from one region of the pair and the rest from the other region.

**Fig. 2:**
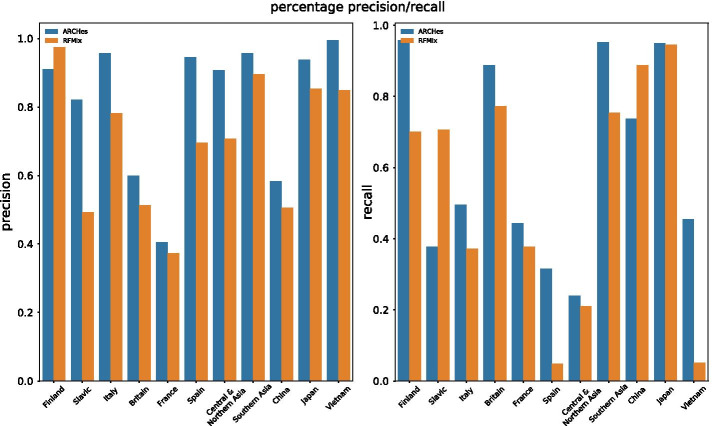
Precision/Recall for each population calculated from estimated ancestry proportions of simulated admixed individuals with ancestry from a pair of neighboring population

We measure precision and recall for each of the 11 unique regions in the set of 16 pairs (Fig. 2). Precision is the amount of correctly identified ancestry divided by the amount estimated for that region and recall is the amount of correctly identified ancestry divided by the total amount of ancestry from that region that is present in the test example. ARCHes outperforms RFMix in terms of *both* precision and recall in eight of the 11 regions, and outperforms it in terms of precision in two more, and in terms of recall in one.

Overall, ARCHes achieves more than 50% global ancestry concordance and diploid local ancestry concordance (Additional file 1: Fig. S3). There is only a small difference between global ancestry concordance and diploid local ancestry concordance on this test set, indicating that ARCHes achieves its global ancestry accuracy by estimating local ancestry accurately. It is also encouraging that ARCHes is capable of differentiating populations not only on a continental level but also on sub-continental and even country levels.

### Separate training and test phases to facilitate high-throughput ancestry estimation

The ARCHes software represents a change in design that explicitly separates two phases, first model creation and annotation and second ancestry estimation, in order to make ancestry estimation both efficient and distributable. The first phase, learning the haplotype models from a large unlabeled training set and then annotating them with the reference panel populations, need only be carried out once. In order to estimate ancestry on subsequent instances, ARCHes software need only reload models and can be run on new examples at any time, distributed as necessary, and the running time depends only on the number of the number of individuals to be processed and labeled, not the size of the reference panels. In contrast, the training and testing processes of RFMix are not separate and require significantly more time per individual. We compare ARCHes's runtime and memory usage with RFMix in Additional file 1: Table S3.

## Discussion

Ancestry inference in large, heterogeneous sample sets is becoming increasingly important for academics, clinicians, and consumers. We developed a new approach, ARCHes, that models ancestry using rich haplotype models coupled to genome-wide information sharing. Our experiments show that ARCHes performs decisively more accurately than a state-of-the-art approach, in terms of both global and local estimation, both within and among continental scales, and among varying levels of admixture. Moreover, because our approach separates the time-consuming training step from the fast testing step, it is well-suited to apply to large scale databases.

Our approach works because haplotypes contain rich information for distinguishing subpopulations, and ARCHes’s haplotype model annotations allow it to quantitatively compare haplotypes to those of several reference panels without requiring that those reference panels be phased, contain haplotypes that are identical to that of an individual, or have similar size or diversity. Indeed, ARCHes can achieve high accuracy with reference panels containing fewer than 50 genotype examples (Additional file 1: Fig. S5). We also note that ARCHes can make use of admixed reference panel members. A genotype example for which we know the diploid (or haploid) population in just a subset of genomic windows can be used in a reference panel to annotate only those windows (though we don’t use this technique in our experiments here).

Our benchmark experiments show that ARCHes is able to capture admixture from a few to several generations removed by learning the genomic scale of admixture on an individual-by-individual basis: more recently admixed samples have relatively longer contiguous blocks of ancestry. This shows that ARCHes is able to be applied broadly without specific, a priori, parameter tuning. This feature is important for analysis of large, heterogeneous databases where it may be difficult to know the specific history of all samples involved.

ARCHes provides a fast and accurate method for inferring unphased local ancestry and combining that into estimates of diploid global ancestry. There are nonetheless several opportunities for future research. First of all, the confidence intervals provided by ARCHes are underestimated; it is possible that they can be improved by using a recalibration procedure on simulated data. Second, despite the fact that using unphased local ancestry in ARCHes helps it to overcome phasing errors, it may be desirable to provide phased local ancestry in some circumstances. Because of the modular nature of the ancestry hidden Markov model, it may be possible to extend this framework to provide phased local ancestry estimates.

## Conclusions

One of the keys to estimating population-level admixture is to measure the similarity between the haplotypes in an individual of unknown origin and those of a reference panel. ARCHes leverages the data from hundreds of thousands of haplotype instances to create haplotype models, and uses a novel approach for employing those models to carry out the comparison. ARCHes can then efficiently estimate population assignments across the genome for large test sets. Our experiments show that across varying amounts of recent admixture, ARCHes outperforms RFMix, a state-of-the-art method in population genetics for local ancestry inference, both in terms of estimating genome-wide population admixture amounts, and at labeling specific genomic regions.

## Methods

### Overall ARCHes method

**Fig. 3:**
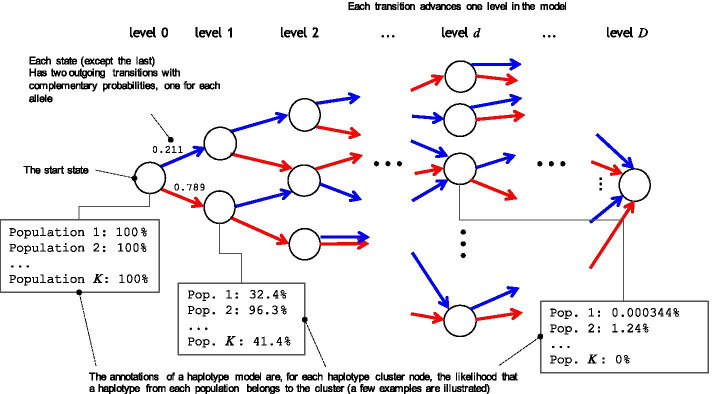
Illustration of annotating haplotype-cluster model representing one genomic window with *D* SNPs (in our experiments *D* is about 75–80, about 3-4 cM). Each box illustrates the expected proportion of haplotypes in all the genotypes of different populations that include a certain model state at a certain level

Our approach begins with dividing the genome into a large number of small windows (e.g., 3–4 centimorgans each), such that, in a recently admixed individual, each of the maternal and paternal haplotypes in a given window are likely to each come from a single population. For each window, we construct a BEAGLE haplotype-cluster model [10] from a large, unlabeled training set of haplotypes. A BEAGLE haplotype-cluster model is a directed acyclic graph with haplotype represented as a path traversing the graph. Each node of the graph represents a cluster of haplotypes. A BEAGLE model is often interpreted as Markov model where the states are the nodes (Additional file 1: Fig. S1), and thus as an “arbitrary order Markov model” of SNPs along a haplotype. Using a reference panel of genotypes from individuals whose ancestry is known in each window, we then annotate each state in the haplotype models with the probability that genotype sequences from a given population belong to the haplotype cluster represented by the state (Fig. 3).

Given a new potentially admixed genotype sequence $x$, we assume that the ancestors of ${\varvec{x}}$ are all ultimately from the $K$ origin groups, and that ${\varvec{x}}$ is admixed recently enough that haplotypes from each group are longer than genomic windows mentioned above, and those haplotypes are much more likely to span an entire window than part of one—i.e., the size of the windows is chosen based on the expected age of admixture. We run a genome-wide hidden Markov model (HMM) whose hidden states are the true assignment (population label pairs) in each window. The emission probabilities are the probability distributions of diploid population assignments for each window arising from the annotated BEAGLE models and the transition probabilities (the probability that the population assignment will change at any point along the genome) are learned through an expectation–maximization (E–M) algorithm. We assign diploid ancestry to each window and estimate the global assignment based on the Viterbi path through this HMM. We also sample paths through the HMM to estimate the uncertainty of assignment amounts.

We describe our detailed method in the following sections, and provide pseudocode in Additional file 1: Appendix S1.

### Annotating haplotype cluster models

We follow Browning and Browning [10] in building haplotype cluster models. Briefly, we divide the genome into *W* partially overlapping windows with approximately the same number of SNPs. Within each window, we build a haplotype cluster model from a large, unlabeled set of training phased haplotypes. For simplicity, we restrict to biallelic variants, and code them as 0 and 1. Building this haplotype cluster model from a large, unlabeled set of individuals provides a “background” of haplotype diversity against which we can measure the informativeness of different haplotypes.

With a haplotype cluster model built for each window, we can then annotate populations using the haplotype cluster model. Recall that each path through a BEAGLE model corresponds to a realization of a haplotype, and each node at a given SNP represents a cluster of haplotypes that are similar near that SNP. For the genotypes of a reference individual in window *w*, **x**_*w*_, we compute the probability that the individual’s two haplotypes pass through two specific nodes in the graph, *u* and *v*, at SNP *d*,

$${P}_{d}\left(u,v|{x}_{w}\right)=\frac{{P}_{d}\left({x}_{w},u,v\right)}{P\left({x}_{w}\right)}$$

where we compute ${P}_{d}\left(u,v|{x}_{w}\right)$ and $P\left({x}_{w}\right)$ using a modification of the forward–backward algorithm for hidden Markov models, treating the node as a hidden state (see Additional file 1: Appendix S1 for pseudocode). In the following, we will refer to the HMM used to analyze the BEAGLE models as the *haplotype HMM*, and its properties as *haplotype emission probabilities,* and *haplotype probabilities*. This contrasts with the *ancestry HMM* we use to smooth ancestry estimates across the genome, which is described in the subsequent section.

We then marginalize over one of the haplotypes of each diploid to create a haplotype posterior probability that the genotypes ${x}_{w}$ in window *w* passes through node *u* at SNP *d*,

$${P}_{d}\left(u|{x}_{w}\right)={\sum }_{v}{P}_{d}\left(u,v|{x}_{w}\right)$$

Finally, we annotate a node *u* by its average haplotype probability in a set of individuals belonging to a reference population *p*, ${R}_{p}=\{{x}_{i,p,w},i\in \mathrm{1,2},\dots ,{n}_{p}\}$ where ${n}_{p}$ is the total number of reference samples in population *p*. Then, we compute

1 $${P}_{d}\left(u|p\right)=\frac{1}{{n}_{p}}{\sum }_{i=1}^{{n}_{p}}{P}_{d}\left(u|{x}_{{\varvec{i}},{\varvec{p}},{\varvec{w}}}\right)$$

This equation gives us the probability that an individual drawn from population *p* will pass through node *u* at SNP *d* of the haplotype cluster model for window *w*.

During the annotation process, we may choose to downsample the genotypes of the reference panel by setting some genotypes at random to ‘missing’ and annotating states of the model by summing over the possible genotypes at those locations. Doing this has the effect of annotating states that represent haplotypes that are similar to those of a reference genotype, but not exactly the same, and is intended to boost performance in reference panels that have few representative examples. We may use the same reference panel individual several times in the annotation process, with a different downsampled genotype each time.

### Ancestry emission probabilities for test individuals in windows

With Eq. (1) in hand, we can compute the probability that a test individual’s genotypes in a given window *w* descend from a specific pair of populations. Letting **t** be the unphased genotype of our test individual, we first compute the probability of **t** given that the two haplotypes in window *w* belong to clusters *u* and *v* of the haplotype cluster model at SNP *d*,

$${P}_{d}\left({\mathbf{t}}_{w}|u,v\right)=\frac{{P}_{d}\left({t}_{w},u,v\right)}{{P}_{d}\left(u,v\right)},$$

where ${P}_{d}\left({\mathbf{t}}_{w},u,v\right)$ is computed using the haplotype forward–backward algorithm and ${P}_{d}\left(u,v\right)$ is obtained by multiplying the transition matrices of the haplotype cluster model up to SNP *d* (equivalent to running the haplotype forward algorithm up to SNP *d* with all haplotype emission probabilities set equal to 1).

We then want to know the probability that the individual’s two haplotypes come from populations *p* and *q* using the information around SNP *d*. We compute this quantity by first computing the probability that a haplotype passes through nodes *u* and *v* and SNP *d* of window *w* given underlying populations *p* and *q* by averaging over the equally likely combinations of whether node *u* corresponds to population *p* and node *v* corresponds to population *q* or vice versa,

#### ${P}_{d}\left(u,v|p,q\right)=\frac{1}{2}\left({P}_{d}\left(u|p\right){P}_{d}\left(v|q\right)+{P}_{d}\left(u|q\right){P}_{d}\left(v|p\right)\right)$.

Note that this result is equivalent to assuming that the two haplotype clusters that make up a diploid sample are independent, and that the two populations that make up those haplotypes are also independent.

Now, we use the law of total probability to average over all haplotype clusters at SNP *d*, and compute the probability that the individual’s haplotype clusters at that point arise from populations *p* and *q*,

#### ${P}_{d}\left({\mathbf{t}}_{w}|p,q\right)={\sum }_{u,v}{P}_{d}\left({\mathbf{t}}_{w}|u,v\right){P}_{d}\left(u,v|p,q\right)$.

This probability weighs similarity to haplotypes in population *p* and *q* more strongly for SNPs closest to SNP *d* in window *w*, because we have no a priori knowledge of which part of a window is most informative about population membership, we finally compute our ancestry emission probability for a window by averaging over the population probability for every SNP in the window,

2 $$P\left({\mathbf{t}}_{w}|p,q\right)=\frac{1}{D}{\sum }_{d}{P}_{d}\left({\mathbf{t}}_{w}|p,q\right)$$

where *D* is the total number of SNPs in window *w*. This process can then be repeated for every window in the genome to obtain the probability of the test individual’s genotype in each window, given that the two haplotypes arose from any pair of populations *p* and *q*.

### Smoothing ancestry estimates using a genome-wide ancestry hidden Markov model

**Fig. 4:**
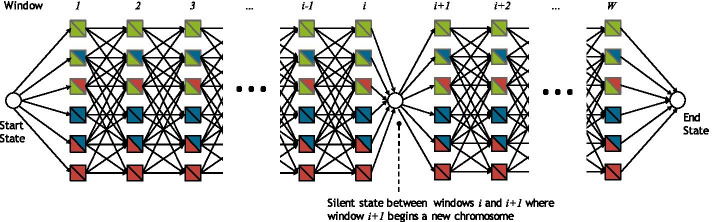
Illustration of genome wide HMM where each window has a series of emitting states, which corresponds to a population assignment *(p,q)* with *1* ≤ *p* ≤ *q* ≤ *K*

In principle, the ancestry emission probabilities computed in the previous section could be used to compute maximum likelihood estimates of diploid local ancestry in each window, one at a time. However, doing so would result in highly noisy ancestry estimates. Instead, we share information across the genome using an additional layer of smoothing via a genome-wide hidden Markov model (Fig. 4). Moreover, because ancestry segments from recent admixture are expected to be longer than a single window, this model helps reduce false ancestry transitions.

If we wish to assign ancestry to $K$ populations, the hidden states of our hidden Markov model are the $\left(\genfrac{}{}{0pt}{}{K}{2}\right)+K$ possible unphased ancestry pairs, $\left(p,q\right)$, with ancestry emission probabilities window *w* given by Eq. (2). Because we model *unphased* diploid ancestry, we define a population pair as unordered, i.e. $\left(p,q\right)$ is the same ancestry assignment as $\left(q,p\right).$ Our ancestry hidden Markov model assumes that between windows ancestry can change for *one* of the two haplotypes with probability $\tau .$ The assumption that ancestry switches only for one of the two haplotypes within an individual is both biologically realistic (assuming individuals are admixed relatively recently) and greatly reduces the complexity of the hidden Markov model. Thus, a change occurs from $\left(p,q\right)$ to $\left({p}^{^{\prime}},{q}^{^{\prime}}\right)$ to any pair such that exactly one of ${p}^{^{\prime}}$ or ${q}^{^{\prime}}$ is different from $p$ or $q$. Each new ancestry pair is drawn with probability proportional to the stationary probability of that ancestry pair,${\pi }_{p,q}$. In full, the transition probabilities are

3 $$P\left( {p^{\prime},q^{\prime}{|}p,q} \right) = \left\{ {\begin{array}{*{20}l} {1 - \tau } \hfill & {if\,p^{\prime} = p,\,q^{\prime} = q} \hfill \\ {\tau \frac{{\pi_{p^{\prime},q^{\prime}} }}{{Z_{p,q} }}} \hfill & {if\,p^{\prime} \ne p, \,q = q^{\prime}\,or\, p^{\prime} = p,\,q^{\prime} \ne \,q} \hfill \\ 0 \hfill & {otherwise} \hfill \\ \end{array} } \right.$$

where the normalizing constant ${Z}_{p,q}$ is given by summing over all accessible unphased haplotype pairs.

Between chromosomes, both ancestry pairs are allowed to change, and the ancestry at the start of each chromosome is drawn independently from that individual’s global distribution of ancestry pairs, ${\pi }_{p,q}$. For a more formal description of how changes between chromosomes are handled, see Additional file 1: Appendix S1.

We initialize the ${\pi }_{p,q}$ to a uniform distribution and $\tau$ to some low value, and use a modified Baum-Welch algorithm to update ${\pi }_{p,q}$ and $\tau$ (see Additional file 1: Appendix S1). Empirically, we observed a tendency to overfit by estimating a large $\tau$ parameter, resulting in inference of a large number of different ancestries; thus we run a fixed number of update steps, rather than stopping at convergence.

### Estimating ancestry proportions in individuals

In principle, the value ${\uppi }_{p}={\sum }_{q}{\uppi }_{p,q}$ could be used as an estimate of the admixture proportion from population $p$ in an individual. However, we instead opt to use a path-based approach that also allows us to obtain credible intervals of the ancestry proportions conditioned on the inferred parameters. Specifically, we provide a point estimate of global ancestry proportions by computing the maximum probability path through the HMM using the Viterbi algorithm, and computing the proportion of windows (weighted by their length) that are assigned to population $p$. We then provide a credible interval by then sampling paths from the posterior distribution on paths, and for each one can compute the ancestry proportion in the same way as from the Viterbi path.

Below we describe experiments we did for benchmarking ARCHes and RFMix [9].

### Reference panel and testing data

We build our reference panel using genotypes from proprietary candidates who explicitly provided prior consent to participate in this research project and have all family lineages tracing back to the same geographic region. All the candidates were genotyped on Ancestry’s SNP array and were analyzed through a quality control pipeline to remove samples with low genotype call rates, samples genetically related to each other, and samples who appear as outliers from their purported population of origin based on Principal Component Analysis. The reference panel contains 11,051 samples, representing ancestry from 32 global regions (Additional file 1: Table S1). We then use 1705 individuals from 1000 Genomes [12] and HGDP Project [13] from 15 populations as testing data. We use SNP array data of individuals from the 1000 Genomes [12] and HGDP [13] projects and limit them to around 300,000 SNPs that overlap with Ancestry’s SNP array. Lists of populations and associated sample counts included in reference panel and testing data are specified in Additional file 1: Tables S1 and S2, respectively. We align populations that come from different data sources, in some cases combining populations together. For example, we combined the ancestries that are assigned to ‘England, Wales, and Northwestern Europe’ and ‘Ireland & Scotland’ to represent ancestry for ‘Britain’. We combined the ancestry that are assigned to ‘Benin & Togo’ and ‘Nigeria’ to represent ancestry for ‘Yoruba’.

### Simulation

We simulate 100 individuals with an admixture history similar to modern Latinos that admixed 12 generations ago with 45% Native American, 50% European and 5% African ancestry. We constructed 100 12-generation pedigrees and randomly selected founders from the reference panel, with the ratio of 45% Native American (from the Maya and Peru regions), 50% European (from the France, Britain, Italy, Spain and Finland regions), and 5% African ancestry (from the Yoruba region). We then simulate the DNA recombination process and obtained the genotypes of the descendant in each pedigree, which are admixed at roughly 45% Native American, 50% European and 5% African.

We simulate genomes of admixed individuals with ancestors from a pair of neighboring populations by simulating genotypes where 1000 Genomes and HGDP test examples serve as the two parents, four grandparents, eight great-grandparents, or 16 great-great-grandparents of a pedigree and the admixed example evaluated is the lone descendant of that set. The examples in this test set are, on average, 50–50% admixed, 25–75% admixed, 12.5–87.5% admixed, or 6.25–93.75% admixed. We simulate 20 individuals for each of the 16 different pairings and 4 different levels of admixture, with half of them representing a minority admixture from one region, and half of them representing a minority admixture from the other region.

Since RFMix requires phased haplotypes for both query and reference individuals, we use Eagle [14] v2 with the HRC [15] reference panel to get phased haplotypes of the simulated individuals as well as for the individuals in the reference panel. However, ARCHes requires only the unphased, diploid genomic sequences for both query and reference individuals.

### RFMix parameters

We first used default parameters in RFMIX v2.03-r0 (https://github.com/slowkoni/rfmix). We then performed a parameter sweep using different number of generations since admixture (the -G parameter), with value of 2, 4, 6 and 8 coupled with different window sizes (set both conditional random field window size and random forest window size) with values of 0.2 cM, 0.5 cM, 100 SNPs (roughly 1 cM) and 300 SNPs (roughly 3 cM) on chromosome 1 of simulated pair admixed individuals. We then selected the parameters with the best performance, namely 4 generations since admixture and a window size 0.2 cM, and ran RFMix on the whole genome of simulated pair admixed individuals. For simulated latino individuals, we used 12 generations since admixture and a window size 0.2 cM. For single origin individuals, we used 2 generations since admixture and a window size 0.2 cM. None of the RFMix runs used the E-M procedure or phase error correction.

Note that for both RFMix and ARCHes, we use the HapMap [16] genetic recombination map for GRCh37 to estimate recombination distance.

### ARCHes parameters

We divide the genome into 3882 windows of 80 SNPs each, overlapping by 5 SNPs (with some adjustments made near chromosome boundaries). We build a haplotype model for each of these windows from a separate cohort of 50,000 haplotypes selected from the Ancestry database that are not already in the population reference panel. We phase these genotypes with Eagle [14], although we do not find that the particular phasing method, or even the diversity of this cohort has a measurable impact on the accuracy of our approach. We tie small groups of 3–4 windows together by disallowing population assignment transitions within those groups, which allows us to set the granularity with which we assign local population assignments (there are 1001 such window groups) and has the benefit of increased computational efficiency. ARCHes's haplotype model annotation process is robust to missing data, which is handled by marginalizing over all possible genotypes. In fact, the annotations may benefit from intentionally downsampling reference panel genotypes so that variations in haplotypes are considered as well, and the amount of downsampling and the number of downsampled genotypes used for annotation are tunable parameters of the annotation process. In our experiments, we sample each reference panel genotype sequence 100 times, each time setting 20% of genotypes to missing and annotating the 3882 haplotype models with them. This training process takes approximately 15 to build each haplotype model and 15 min to annotate it, although that process is parallelizable and need not be carried out again, regardless of the size of the test set. We set the initial *τ*_x_ parameter to be 0.01 and learned this parameter using 10 iterations of the E-M approach described above. ARCHes assigns diploid local ancestry to 1001 windows of the genome and the global ancestry estimates are summarized from these 1001 windows.

## Supplementary Information

**Additional file 1: Supplementary Materials**. Contains Figures S1–S5, Tables S1 and S2, and Appendix S1 which contains implementation details, formulas, and pseudocode.

**Additional file 2: Data Tables**. A spreadsheet containing 11 tables, showing test set labels, population estimates for test set examples, precision and recall by population, single-origin confusion matrices (for ARCHes and RFMix), global and local average concordance for the paired-population test sets, their corresponding raw concordance scores, global concordance for the simulated Latino test sets, local concordance for the simulated Latino test sets, Fst statistics for the paired populations in test sets, and Fst statistics between reference panels and test sets.

## Data Availability

All data generated for this study are available in Additional file 2. Data used in this study from the 1000 Genomes Project data are available at https://www.internationalgenome.org. Data used in this study from the Human Genome Diversity Project (HGDP) are available at https://www.hagsc.org/hgdp/. Individual genotype data for human subjects participating in Ancestry DNA’s Human Diversity Project are not available, to protect their privacy and anonymity.

## Abbreviations

ARCHes: Ancestry inference using Reference labeled Clusters of Haplotypes
cM: Centimorgan(s)
HGDP: Human Genome Diversity Project
HMM: Hidden Markov model
HRC: Haplotype reference consortium
E–M: Expectation–maximization
SNP: Single nucleotide polymorphism
