# Supplementary material for: Ancestry inference using reference labeled clusters of haplotypes
Source: BMC Bioinformatics. 2021 Sep 25;22:459. doi: 10.1186/s12859-021-04350-x (PMC8466715; doi:10.1186/s12859-021-04350-x)
Supplement: Supplementary file 1 — Additional file 1: Supplementary Materials. Contains Figures S1–S5, Tables S1 and S2, and Appendix S1 which contains implementation details, formulas, and pseudocode. [file 12859_2021_4350_MOESM1_ESM.pdf]

Supplementary Materials

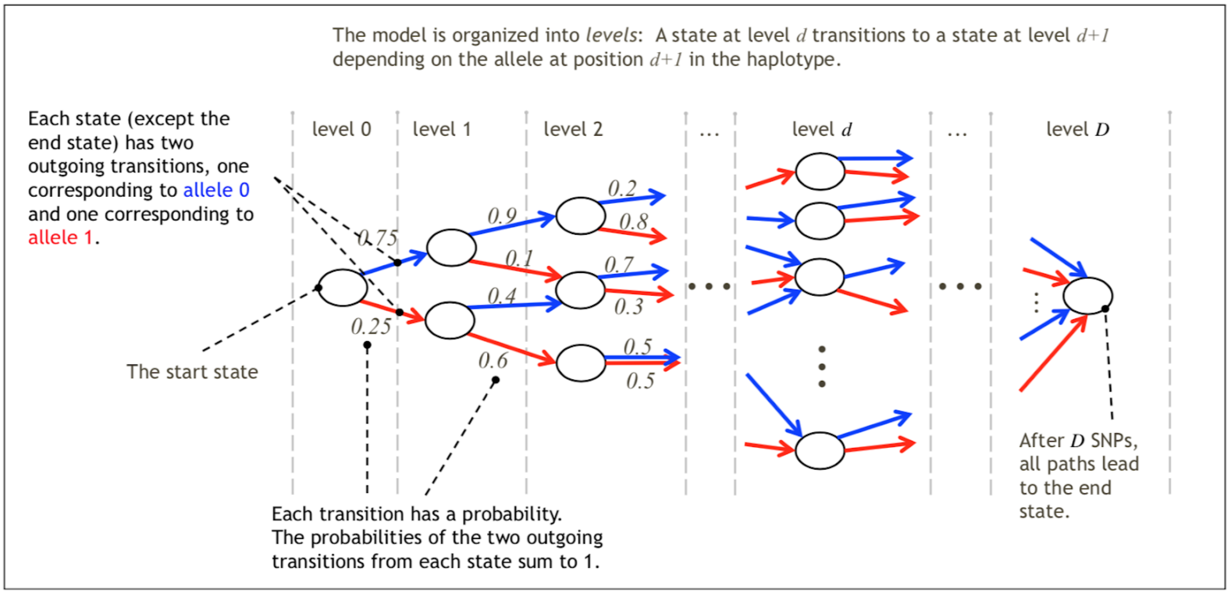

Fig. S1. Illustration of haplotype model for one window of the genome, consisting of *D* SNPs.

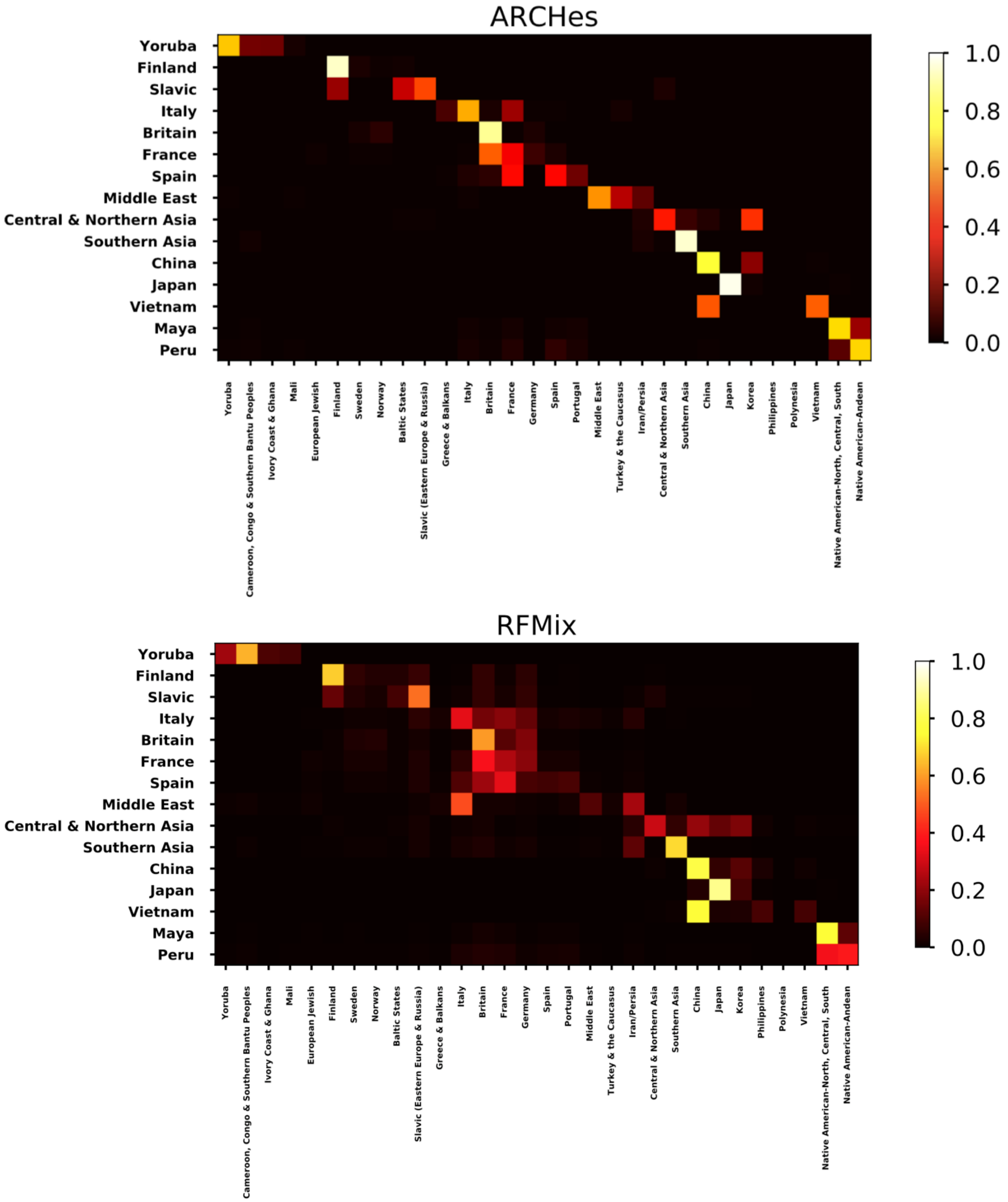

Fig. S2. Average estimated ancestry proportions for single-origin individuals from each testing population. In this matrix figure, each row represents single-origin individuals from the testing population. Each column represents each of the possible 30 populations that the single-origin individuals might be assigned to.

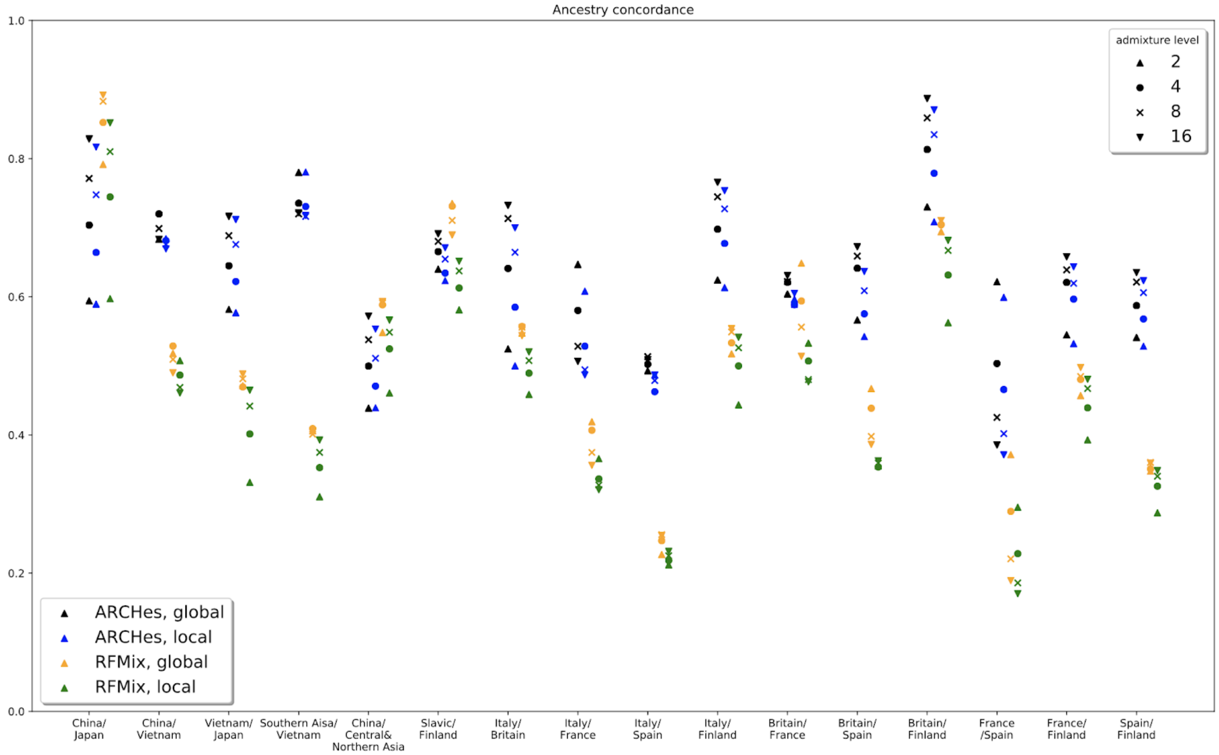

Fig. S3. Concordance of global ancestry assignments and diploid local ancestry assignments for simulated admixed individuals from 16 different pairings of 11 populations. Admixture level 2 means each of two parents belongs to different populations (50%-50% admixture), admixture level 4 means one of four grandparents belongs to one population and the other three belong to the other population (roughly 25%-75% admixture), admixture level 8 means one of eight great-grandparents belongs to the first population (12.5%-87.5%), and level 16 means one of sixteen great-great-grandparents belong to the first population (6.25%-93.75%).

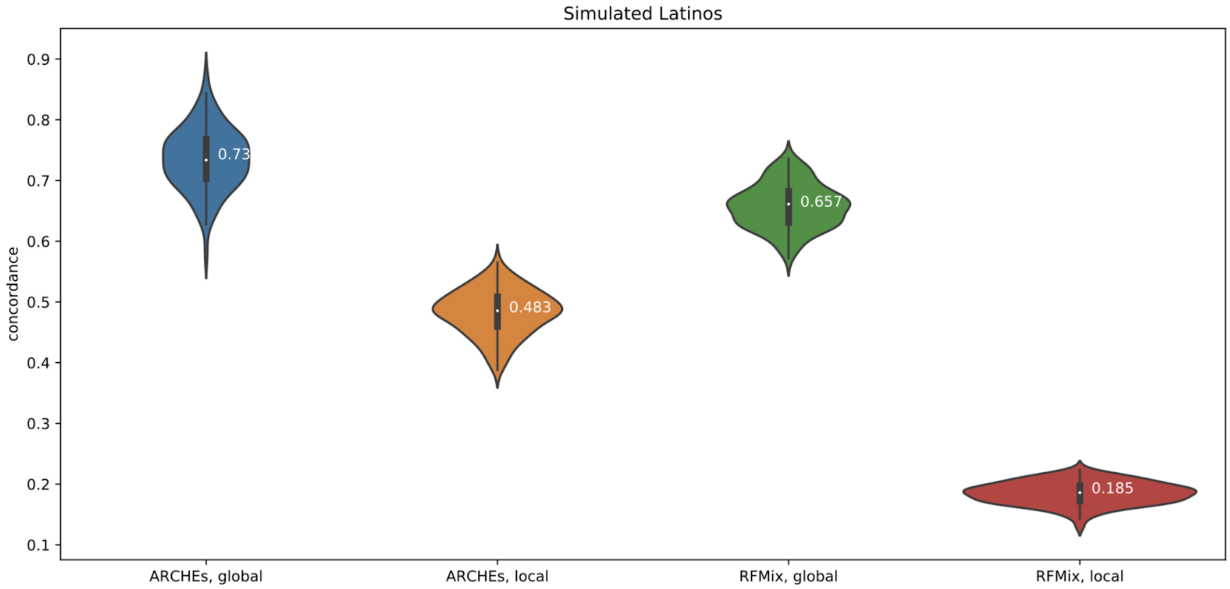

Fig. S4. Concordance of global ancestry assignments and diploid local ancestry assignments on 100 simulated Latino individuals.

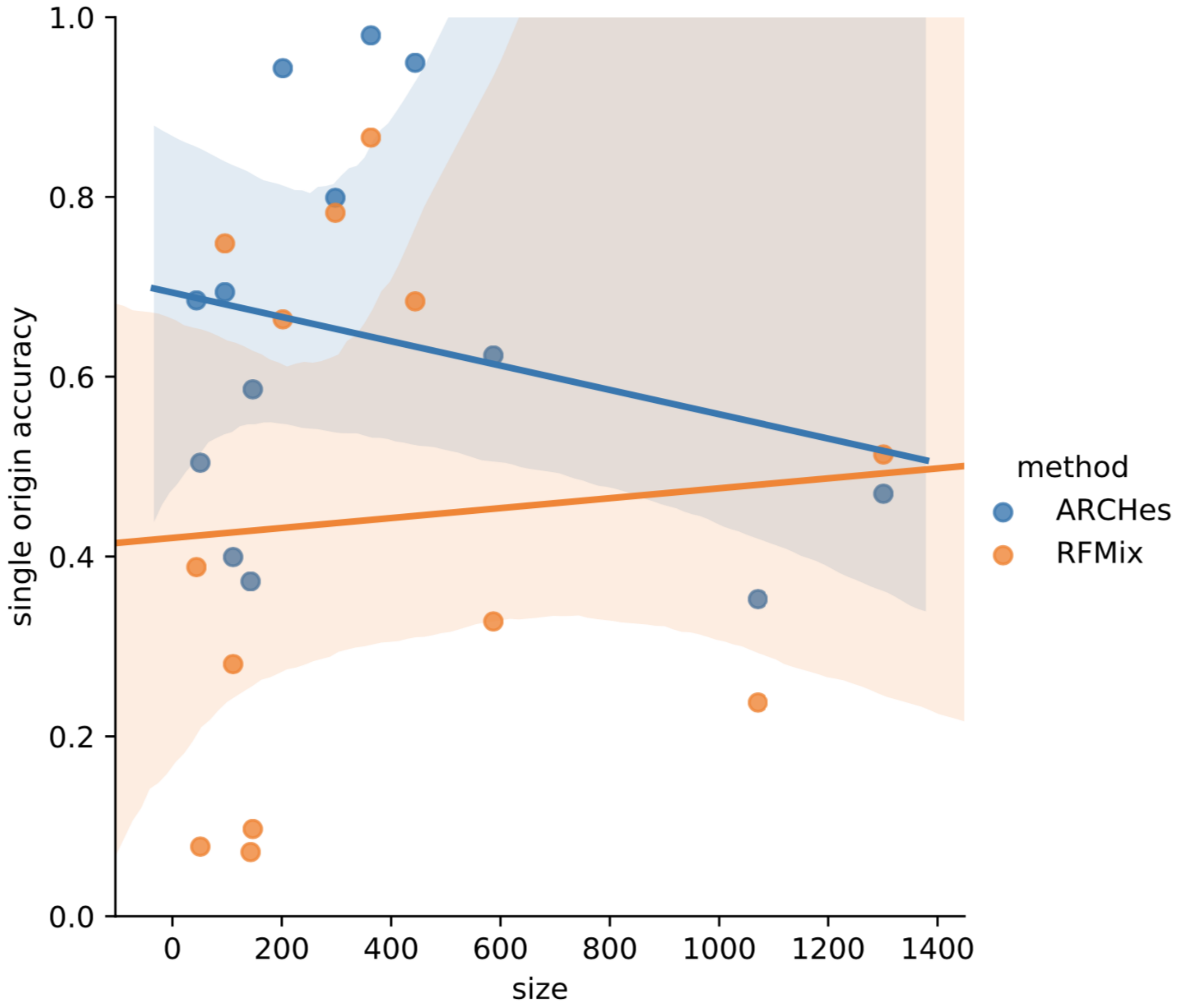

Fig. S5. Relationship between the number of individuals in the reference panel and the accuracy for single origin individuals for each population.

Table S1. Sample size and geographic location for 32 populations in the reference panel. Some populations are matched with testing populations specified in Supplemental Table 2.

Population Label	Sample size	Matched testing population
Native American-North, Central, South	96	Maya
Native American-Andean	44	Peru
England, Wales, and Northwestern Europe	1226	Britain
Central & Northern Asia	111	Central & Northern Asia
Southern Asia	444	Southern Asia
Baltic States	127	
Benin & Togo	102	Yoruba
Cameroon, Congo & Southern Bantu Peoples	576	
Ireland & Scotland	319	Britain
China	298	China
European Jewish	129	
France	1071	France
Germany	1314	
Greece & Balkans	149	
Italy	587	Italy
Ivory Coast & Ghana	119	
Japan	363	Japan
Korea	201	
Mali	169	
Middle East	147	Middle East
Nigeria	109	Yoruba

Norway	242	
Iran/Persia	413	
Philippines	385	
Polynesia	57	
Portugal	257	
Slavic (Eastern Europe & Russia)	1301	Slavic
Spain	143	Spain
Sweden	240	
Turkey & the Caucasus	59	
Finland	202	Finland
Vietnam	51	Vietnam

Table S2. Sample size and geographic label for testing population from HGDP and 1000 Genomes.

Population label	Detailed label	Sample size	Source
Maya	Maya	25	HGDP
Peru	PEL(Peruvians from Lima, Peru)	105	1000 Genomes
Central & Northern Asia	Daur, Hazara, Hezhen, Mongola, Oroqen, Tu, Uygur, Xibo, Yakut	116	HGDP
Southern Asia	Pathan, Sindhi	48	HGDP
Yoruba	YRI (Yoruba in Ibadan, Nigeria), Yoruba	213	1000 Genomes, HGDP
China	CHS (Southern Han Chinese), Han, She, Tujia	325	1000 Genomes, HGDP
France	French	29	HGDP
Britain	GBR (British in England and Scotland)	104	1000 Genomes
Italy	TSI (Toscani in Italia)	112	1000 Genomes
Japan	JPT (Japanese in Tokyo, Japan), Japanase	134	1000 Genomes, HGDP
Middle East	Druze, Palestinian	98	HGDP
Slavic	Russian	25	HGDP
Spain	IBS (Iberian Population in Spain)	150	1000 Genomes
Finland	FIN (Finnish in Finland)	100	1000 Genomes
Vietnam	KHV (Kinh in Ho Chi Minh City, Vietnam)	121	1000 Genomes

Table S3. Run time and Memory Usage (Maximum resident set size, MaxRSS) comparison between ARCHes and RFMix. Since ARCHes trains models in a separate process, we only count the running time and MaxRSS for inferring ancestry for test individuals. However, because RFMix combines the training and testing process together, we count the running time and memory use (MaxRSS) for both training and testing process for RFMix.

Experiment	# of test individuals	Method	User time (s)	MaxRSS
Single origin individual	1705	ARCHes	98237 (10 CPU)	7.9G
		RFMix	390443 (10 CPU)	6.18G
Simulated pair admixed individual	3200	ARCHes	188709 (10 CPU)	14.8G
		RFMix	378838 (10 CPU)	7.27G
Simulated Latino individual	100	ARCHes	6814 (1 CPU)	0.53G
		RFMix	389388 (10 CPU)	8.07G

Appendix S1

A Implementation of BEAGLE Haplotype Models

The haplotype models we annotate and use to compute the ancestry HMM emission probabilities are BEAGLE [BB07] haplotype models, but they must be computed once and written to disk, and differ from the BEAGLE implementation in the following ways.

First, the transition probabilities are based on the haplotype counts observed in the training set, but smoothed so that all possible haplotypes have a nonzero probability. Specificially, the transition probability from a haplotype cluster corresponding to allele a is

$$P(a) = \frac{1}{2}\gamma + \frac{n_a}{n_a + n_{\bar{a}}}(1-\gamma) \tag{4}$$

where n_a is the count of haplotypes in the cluster with allele a and $n_{\bar{a}}$ is the count with the alternative allele, and γ is a user-specified weight (we set $\gamma = 10^{-4}$).

Second, because we build large haplotype models from hundreds of thousands of training examples, and the diploid-HMMs are quadratically larger than the haplotype models, we discard a portion of the lowest-probability states in the diploid-HMM state space after each step in the forward procedure, in order to make the procedure more efficient. Specifically, after computing the set of states (*i.e.,* possible pairs of haplotype clusters) for each SNP d, we sort states by forward probability and discard the least likely states, but no more than a small proportion ϵ (we set $\epsilon = 10^{-6}$) of the probability mass at level d.

Third, in the haplotype model building procedure, we decide when to merge two haplotype clusters based on slightly different criteria than BEAGLE. Let n_x and n_y be the respective number of haplotypes in haplotype clusters X and Y, and let $n_x(h)$ and $n_y(h)$ be the observed occurrences of a haplotype h in X and Y, respectively. The frequency of h in X and Y is estimated to be $\hat{p}_x^{(h)} = \frac{n_x(h)}{n_x}$ and $\hat{p}_y^{(h)} = \frac{n_y(h)}{n_y}$, respectively. BEAGLE will not merge two clusters if

$$\left|\hat{p}_x^{(h)} - \hat{p}_y^{(h)}\right| \geq \sqrt{\frac{1}{n_x} + \frac{1}{n_y}}. \tag{5}$$

We reformulate the inequality as a hypothesis test based on Welch's t-test [Wel47] which is, in the form of the inequalities above,

$$\left|\hat{p}_x^{(h)} - \hat{p}_y^{(h)}\right| \geq C\sqrt{\frac{\hat{p}_x^{(h)}(1-\hat{p}_x^{(h)})}{n_x} + \frac{\hat{p}_y^{(h)}(1-\hat{p}_y^{(h)})}{n_y}} \tag{6}$$

where C is a pre-defined constant. The concern with Welch's t-test is that it is too confident in its estimation of variance when the frequency estimate is close to 0 or 1. To avoid this problem, we regularize the frequency estimate using a symmetric beta distribution as a prior. Thus, we replace the $\hat{p}$ estimates with their posteriors:

$$\tilde{p}_x^{(h)} = \frac{n_x(h) + \alpha}{n_x + \alpha + \beta} \tag{7}$$

$$\tilde{p}_y^{(h)} = \frac{n_y(h) + \alpha}{n_y + \alpha + \beta} \tag{8}$$

and rewrite (6) as

$$\left|\tilde{p}_x^{(h)} - \tilde{p}_y^{(h)}\right| \geq C\sqrt{\frac{\tilde{p}_x^{(h)}(1-\tilde{p}_x^{(h)})}{n_x} + \frac{\tilde{p}_y^{(h)}(1-\tilde{p}_y^{(h)})}{n_y}} \tag{9}$$

or equivalently

$$\frac{(\tilde{p}_x^{(h)} - \tilde{p}_y^{(h)})^2}{\frac{\tilde{p}_x^{(h)}(1-\tilde{p}_x^{(h)})}{n_x} + \frac{\tilde{p}_y^{(h)}(1-\tilde{p}_y^{(h)})}{n_y}} \geq C^2. \tag{10}$$

We use $\alpha = \beta = \frac{1}{2}$ and $C^2 = 20$.

B Pseudocode for Diploid HMM Forward and Backward Procedures

Algorithm 1 Diploid HMM forward procedure for a sequence $\mathbf{x}$ of D diploid genotypes (values are all homozygous 0 or 1, heterozygous, or missing) and a model $\mathbf{M}$ of $D+1$ levels. $\mathbf{M}$ has a start state $\mathbb{S}$, a transition function $t(u,a)$ that maps a haplotype model state u to the state at the next level associated with the allele a transition ($a \in 0,1$), and a transition probabilty function $\rho(u,a)$ that maps a haplotype model state u to the transition probability associated with allele a. The procedure populates f, where $f(u_1,u_2)$ is the forward likelihood of a diploid HMM state (u_1,u_2). It also stores states of the diploid HMM that are consistent with the genome at each level and their outgoing transitions (and the probabilities associated with those transitions) to a data structure α (so that the genotype need not be re-examined during the backward procedure). The *optional* subroutine TRIM removes the diploid HMM states in a set with the lowest f values. It is often possible to remove a large proportion of states and yet keep (*e.g.,*) 99.9999% of the likelihood mass contained in the set of SNPs. We use TRIM only for reasons of efficiency.

procedure DIPLOID-FORWARD($\mathbf{x}, w, \mathbf{M}_w$)
  Let D_w be the number of SNPs in $\mathbf{M}_w$
  Let $\mathcal{W}(\mathbf{x}, w)$ be the subsequence of genotypes in $\mathbf{x}$ that correspond to the SNPs in window w.
  Let $\mathbb{S}$ be the start state of model $\mathbf{M}_w$
  Let t and ρ be $\mathbf{M}_w$'s transition functions, mapping a state to a state and probability, respectively
  Let $\alpha(d)$ be an initially empty data structure containing diploid HMM states at level d,
    and the states they transition to with what probability
  $f(\mathbb{S},\mathbb{S})) \leftarrow 1$ *// both haplotypes must start in the haplotype model start state*
  Add state $(\mathbb{S},\mathbb{S})$ to $\alpha(0)$ with no outgoing transitions (yet)
  for $d \in 0,1,2,..., D_w - 1$ **do**
    for each diploid HMM state $(u_1,u_2) \in \alpha(d)$ **do**
      Let P be an initially empty list of diploid HMM state transitions and their likelihoods
      if $\mathcal{W}(\mathbf{x}, w)_{d+1}$ is HOMOZYGOUS 0 **then**
        Add $((t(u_1,0), t(u_2,0)), \rho(u_1,0) \times \rho(u_2,0))$ to P
      if $\mathcal{W}(\mathbf{x}, w)_{d+1}$ is HOMOZYGOUS 1 **then**
        Add $((t(u_1,1), t(u_2,1)), \rho(u_1,1) \times \rho(u_2,1))$ to P
      if $\mathcal{W}(\mathbf{x}, w)_{d+1}$ is HETEROZYGOUS **then** *// Consider both possibilities*
        Add $((t(u_1,0), t(u_2,1)), \rho(u_1,0) \times \rho(u_2,1))$ to P
        Add $((t(u_1,1), t(u_2,0)), \rho(u_1,1) \times \rho(u_2,0))$ to P
      if $\mathcal{W}(\mathbf{x}, w)_{d+1}$ is MISSING **then** *// Consider all possibilities*
        Add $((t(u_1,0), t(u_2,0)), \rho(u_1,0) \times \rho(u_2,0))$ to P
        Add $((t(u_1,0), t(u_2,1)), \rho(u_1,0) \times \rho(u_2,1))$ to P
        Add $((t(u_1,1), t(u_2,0)), \rho(u_1,1) \times \rho(u_2,0))$ to P
        Add $((t(u_1,1), t(u_2,1)), \rho(u_1,1) \times \rho(u_2,1))$ to P
      for $((v_1,v_2),p)$ in P **do** *// (u_1,u_2) can transition to (v_1,v_2) with probability p*
        if (v_1,v_2) is not in $\alpha(d+1)$ **then** *// Lookup in constant time with perfect hash on serial numbers of v_1,v_2*
          initialize $f(v_1,v_2) \leftarrow 0$ and add (v_1,v_2) to $\alpha(d+1)$
        $f(v_1,v_2) \leftarrow f(v_1,v_2) + f(u_1,u_2) \times p$ *// Update $f(v_1,v_2)$ to include the new transition*
        Add $((u_1,u_2) \rightarrow (v_1,v_2), p)$ to the set of outgoing transitions for state (u_1,u_2) in $\alpha(d)$
    TRIM$(\alpha(d+1), f)$ *// Optionally remove some of the lowest-likelihood diploid HMM states from $\alpha(d+1)$*
  return f, α

Algorithm 2 Diploid HMM backward procedure (see Diploid-Forward). The procedure populates b, where $b(u_1, u_2)$ is the backward likelihood of a diploid HMM state (u_1, u_2). D is the number of SNPs in the window associated with the haplotype model, α is the set of diploid HMM states at each level and their probabilistic outgoing transitions as computed by Diploid-Forward.

procedure Diploid-Backward(D, α)
  Initialize $b(u_1, u_2) \leftarrow 0$ for all diploid HMM states (u_1, u_2)
  for $d \in D-1, D-2, ..., 2, 1, 0$ **do**
    for each diploid HMM state $(u_1, u_2) \in \alpha(d)$ **do** *// (u_1, u_2) is a source state*
      for each diploid HMM state (v_1, v_2) such that $((u_1, u_2) \rightarrow (v_1, v_2), p) \in \alpha(d)$ **do** *// (v_1, v_2) is a destination state*
        // (u_1, u_2) transitions to (v_1, v_2) with probability p
        $b(u_1, u_2) \leftarrow b(u_1, u_2) + b(v_1, v_2) \times p$
  return b

Algorithm 3 Diploid HMM forward-backward procedure (see Diploid-Forward and Diploid-Backward). The procedure populates f and b, where $f(u_1, u_2)$ is the ("forward") likelihood that a path through the diploid HMM ends in state (u_1, u_2) after emitting d alleles (where d is the level of u_1 and u_2) of a haplotype in the input genotype sequence $\mathbf{x}$, and $b(u_1, u_2)$ is the likelihood of all paths from (u_1, u_2) to the end state. The probability $P_d(u_1, u_2|\mathbf{x})$ that the haplotypes of genotype sequence $\mathbf{x}$ belongs to clusters u_1 and u_2 is calculated as $\frac{f(u_1,u_2)b(u_1,u_2)}{b(\mathbb{S},\mathbb{S})}$, where $\mathbb{S}$ is the start state of model $\mathbf{M}$ and f and b are computed by this procedure.

procedure Diploid-Forward-Backward$(\mathbf{x}, w, \mathbf{M}_w)$
  $f, \alpha \leftarrow$Diploid-Forward$(\mathbf{x}, w, \mathbf{M}_w)$
  Let D_w be the number of SNPs in $\mathbf{M}_w$
  $b \leftarrow$Diploid-Backward(D_w, α)
  return f, b

C Computing Forward-Backward on the Genome-Wide Ancestry HMM, and Updating π and τ Transition Probability Parameters

The genome-wide ancestry HMM computes the likelihoods that a test instance's genotype sequence, $\mathbf{t}$, in a genomic window w (denoted $\mathbf{t}_w$) is explained by populations p and q for a set of populations and genomic windows. It is parameterized by $\pi_{\mathbf{t}}$ and $\tau_{\mathbf{t}}$,[1] which are typically learned for a specific test instance. The ancestry HMM representing a K populations and a set of SNPs on multiple chromosomes has a single silent (non-emitting) state before the first, after the last, and in-between each chromosome, and a series of $\frac{(K+1)\times K}{2}$ emitting states for each window of each chromosome, each corresponding to a population assignment (p, q) with $1 \leq p \leq q \leq K$. Figure 4 illustrates such a genome-wide HMM with $K = 3$ populations. Let the emitting state corresponding to window w and population assignment (p, q) be denoted $S_{w,p,q}$. Its emission probability $P(\mathbf{t}_w|p, q)$ is precomputed and fixed based on the genotype $\mathbf{t}_w$ in window w. Let S_c represent the silent state that preceeds the emitting states correpsonding to windows on chromosome c. Thus the start state of the HMM is S_1 (and if the HMM represents C chromosomes, the end state would be S_{C+1}). Let $\mathcal{C}(c)$ map a chromosome number to the window that begins the chromosome. Then, our HMM transitions from silent states to emitting states $S_c \rightarrow S_{\mathcal{C}(c),p,q}$, from emitting states in the last window of a chromosome to a silent state $S_{\mathcal{C}(c+1)-1,p,q} \rightarrow S_{c+1}$, and from emitting states to emitting states for windows w that are not the first or last in a chromosome $S_{w,p,q} \rightarrow S_{w+1,p',q'}$. A transition from $S_{w,p,q} \rightarrow S_{w+1,p',q'}$ represents a change in population assignment between windows w and $w+1$ if $p \neq p'$ or $q \neq q'$.

The transition probabilities from a silent state to an emitting state $S_c \rightarrow S_{\mathcal{C}(c),p,q}$ is $\pi_{\mathbf{t},(p,q)}$, where $\pi_{\mathbf{t}}$ is a learned parameter vector over all possible assignments (p, q) $(1 \leq p \leq q \leq K)$ indicating a global assigment preference. The transition probability from a state in the last window of a chromosome to a silent state $S_{\mathcal{C}(c)-1,p,q} \rightarrow S_c$ is always 1, and transitions between emitting states on the same chromosome, from state

[1]The subscript $\mathbf{t}$ may be dropped from these and other terms when there is only one test genotype instance in question.

(p, q) in window w to state (p', q') (with $p' \leq q'$) in window $w + 1$, are as follows:

$$P(S_{w,p,q} \to S_{w+1,p',q'} | \pi_{\mathbf{t}}, \tau_{\mathbf{t}}) = \begin{cases} 1 - \tau_{\mathbf{t}} & \text{if } p = p' \text{ and } q = q' \\ \tau_{\mathbf{t}} \times \frac{\pi_{\mathbf{t},(p',q')}}{\sum_{(p'',q'')|p'' \leq q'', p = p'' \oplus q = q''} \pi_{\mathbf{t},(p'',q'')}} & \text{if } p = p' \oplus q = q' \\ 0 \text{ (transition ignored)} & \text{otherwise} \end{cases} \tag{11}$$

where $\tau_{\mathbf{t}}$ is a parameter representing the probability of changing population assignment that enforces the bias against changing population assignments from window to window ($\oplus$ is the exclusive or operator). We initialize $\pi_{\mathbf{t}}$ to a uniform distribution, and $\tau_{\mathbf{t}}$ to a (typically low) initial value and learn $\pi_{\mathbf{t}}$ and $\tau_{\mathbf{t}}$ using expectation-maximization over a number of iterations (similar to the standard Baum-Welch algorithm [Rab89], except that $\pi_{\mathbf{t}}$ and $\tau_{\mathbf{t}}$ are tied to all state transition probabilities).

Let $F_{\mathbf{t}}(s)$ be the forward probability, the sum probability of all paths through the Ancestry HMM (as opposed to the *haplotype* HMM used to calculate per-window emission probabilities) that start in the start state and end in state s (including the emission of state s) and $B_{\mathbf{t}}(s)$ be the backward probability of all paths through the HMM that start in state s (excluding emission) and end in the end state. F and B are computed recursively as follows.

$$F_{\mathbf{t}}(S_1) = 1. \tag{12}$$

For the emitting states in the first window of a chromosome,

$$F_{\mathbf{t}}(S_{\mathcal{C}(c),p',q'}) = F_{\mathbf{t}}(S_c) \times \pi_{\mathbf{t},(p',q')} \times P(\mathbf{t}_{\mathcal{C}(c)}|p, q) \tag{13}$$

for all p' and q'. When a window w is not the first window of a chromosome,

$$F_{\mathbf{t}}(S_{w,p',q'}) = \sum_{p=1}^{K} \sum_{q=p}^{K} F_{\mathbf{t}}(S_{w-1,p,q}) \times P(S_{w-1,p,q} \to S_{w,p',q'} | \pi_{\mathbf{t}}, \tau_{\mathbf{t}}) \times P(\mathbf{t}_w | p, q) \tag{14}$$

where $P(S_{w-1,p,q} \to S_{w,p',q'} | \pi_{\mathbf{t}}, \tau_{\mathbf{t}})$ is given by (11). The forward probability of the silent state preceding chromosome c is

$$F_{\mathbf{t}}(S_c) = \sum_{p=1}^{K} \sum_{q=p}^{K} F_{\mathbf{t}}(S_{\mathcal{C}(c)-1,p,q}) \tag{15}$$

Similarly, if there are C chromosomes in the model,

$$B_{\mathbf{t}}(S_{C+1}) = 1. \tag{16}$$

For the last window on chromosome c,

$$B_{\mathbf{t}}(S_{\mathcal{C}(c+1)-1,p,q}) = B_{\mathbf{t}}(S_{c+1}) \tag{17}$$

for all p and q. When window w is not the last window on a chromosome,

$$B_{\mathbf{t}}(S_{w,p,q}) = \sum_{p'=1}^{K} \sum_{q'=p'}^{K} P(S_{w,p,q} \to S_{w+1,p',q'} | \pi_{\mathbf{t}}, \tau_{\mathbf{t}}) \times P(\mathbf{t}_{w+1} | p', q') \times B_{\mathbf{t}}(S_{w+1,p',q'}). \tag{18}$$

Finally, for the silent state preceding chromosome c,

$$B_{\mathbf{t}}(S_c) = \sum_{p'=1}^{K} \sum_{q'=p'}^{K} \pi_{\mathbf{t},p',q'} \times P(\mathbf{t}_{\mathcal{C}(c)} | p', q') \times B_{\mathbf{t}}(S_{\mathcal{C}(c),p',q'}). \tag{19}$$

After computing $\mathbf{F_t}$ and $\mathbf{B_t}$, we compute the expectation for each $\pi_{\mathbf{t},(p,q)}$ as

$$\mathbf{E}(\pi_{\mathbf{t},(p,q)}) = \sum_{c=1}^{C} \sum_{w=\mathcal{C}(c)}^{\mathcal{C}(c+1)-1} F_{\mathbf{t}}(w,p,q) \times B_{\mathbf{t}}(w,p,q) \tag{20}$$

and reset each $\pi_{\mathbf{t},(p,q)}$ to the value that maximizes the likelihood of $\mathbf{E}(\pi_{\mathbf{t},(p,q)})$:

$$\pi_{\mathbf{t},(p,q)} \leftarrow \frac{\mathbf{E}(\pi_{\mathbf{t},(p,q)})}{\sum_{p'=1}^{K} \sum_{q'=p'}^{K} \mathbf{E}(\pi_{\mathbf{t},(p',q')})}. \tag{21}$$

We learn $\tau_{\mathbf{t}}$ in a similar fashion, by updating it based on the expected number of transitions that do not change assignment, compared to all transitions. If there are C chromosomes,

$$\tau_{\mathbf{t}} \leftarrow 1 - \frac{\sum_{c=1}^{C} \sum_{w=\mathcal{C}(c)}^{\mathcal{C}(c+1)-1} \sum_{p=1}^{K} \sum_{q=p}^{K} F_{\mathbf{t}}(S_{w,p,q}) \times P(S_{w,p,q} \rightarrow S_{w+1,p,q}|\pi_{\mathbf{t}}, \tau_{\mathbf{t}}) \times B_{\mathbf{t}}(S_{w+1,p,q})}{\sum_{c=1}^{C} \sum_{w=\mathcal{C}(c)}^{\mathcal{C}(c+1)-1} \sum_{p=1}^{K} \sum_{q=p}^{K} \sum_{p'=1}^{K} \sum_{q'=p'}^{K} F_{\mathbf{t}}(S_{w,p,q}) \times P(S_{w,p,q} \rightarrow S_{w+1,p',q'}|\pi_{\mathbf{t}}, \tau_{\mathbf{t}}) \times B_{\mathbf{t}}(S_{w+1,p',q'})} \tag{22}$$

D Computing the Viterbi Path

The Viterbi path is the single most likely path (relative to a genotype sequence $\mathbf{t}$) through the genome-wide HMM $\mathbf{V_t} = \langle V_{\mathbf{t},1}, V_{\mathbf{t},2}, ..., V_{\mathbf{t},W} \rangle$, where each $V_{\mathbf{t},w}$ is an assignment (p,q) in a window w, $1 \leq w \leq W$.

To compute $\mathbf{V}$, we must first define $\mathbf{M_t}$, where $M_{\mathbf{t}}(s)$ is the probability of the most likely path through the HMM that start in the start state and end in state s (including the emission of state s), analagous to the forward probability $F_{\mathbf{t}}(s)$ AppendixC, but referring to the probability of the single most likely path instead of the sum probability of all paths.

$$M_{\mathbf{t}}(S_1) = 1. \tag{23}$$

For the emitting states in the first window of a chromosome,

$$M_{\mathbf{t}}(S_{\mathcal{C}(c),p',q'}) = M_{\mathbf{t}}(S_c) \times \pi_{\mathbf{t},(p',q')} \times P(\mathbf{t}_{\mathcal{C}(c)}|p',q') \tag{24}$$

for all p' and q'. When a window w is not the first window of a chromosome,

$$M_{\mathbf{t}}(S_{w,p',q'}) = \operatorname*{argmax}_{1 \leq p \leq q \leq K} M_{\mathbf{t}}(S_{w-1,p,q}) \times P(S_{w-1,p,q} \rightarrow S_{w,p',q'}|\pi_{\mathbf{t}}, \tau_{\mathbf{t}}) \times P(\mathbf{t}_w|p',q') \tag{25}$$

And for a silent state that is not the start state,

$$M_{\mathbf{t}}(S_c) = \operatorname*{argmax}_{1 \leq p \leq q \leq K} M_{\mathbf{t}}(S_{\mathcal{C}(c)-1,p,q}) \tag{26}$$

The Viterbi path $\mathbf{V}$ is then defined for windows that are the last window in a chromosome, c, as

$$V_{\mathbf{t},\mathcal{C}(c+1)-1} = \operatorname*{argmax}_{1 \leq p \leq q \leq K} M_{\mathbf{t}}(S_{\mathcal{C}(c+1)-1}, p, q), \tag{27}$$

and for all other windows as

$$V_{\mathbf{t},w} = \operatorname*{argmax}_{1 \leq p' \leq q' \leq K} P(S_{w,p,q} \rightarrow S_{w+1,p',q'}|\pi_{\mathbf{t}}, \tau_{\mathbf{t}}) \times P(\mathbf{t}_{w+1}|p',q') \times M_{\mathbf{t}}(S_{w+1,p',q'}). \tag{28}$$

E Computing Path Samples

Let *choose* be a operator that chooses an argument with a probability relative to an expression so that $\underset{x \in D}{\text{choose}}\, f(x)$ returns x with probability $\frac{f(x)}{\sum_{x' \in D} f(x')}$. Then a stochastic path $\mathbf{Q}$ for a genomic sequence $\mathbf{t}$ is defined over all windows $1 \leq w \leq W$ as follows. For windows that are last in a chromosome, c,

$$Q_{\mathbf{t},\mathcal{C}(c+1)-1} = \underset{p,q}{\text{choose}}\, F_{\mathbf{t}}(S_{\mathcal{C}(c+1)-1,p,q}). \tag{29}$$

For other windows w,

$$Q_{\mathbf{t},w} = \underset{p,q}{\text{choose}}\, F_{\mathbf{t}}(S_{w,p,q}) \times P(S_{w,p,q} \rightarrow S_{w+1,Q_{\mathbf{t},w+1}} | \pi_{\mathbf{t}}, \tau_{\mathbf{t}}) \times P(\mathbf{t}_{w+1} | Q_{\mathbf{t},w+1}). \tag{30}$$

References

[BB07] Sharon R. Browning and Brian L. Browning. Rapid and accurate haplotype phasing and missing-data inference for whole-genome association studies by use of localized haplotype clustering. *American Journal of Human Genetics*, 81:1084–1096, 2007.

[Rab89] L. Rabiner. A tutorial on hidden Markov models and selected applications in speech recognition. *Proceedings of the IEEE*, 77(2):257–286, 1989.

[Wel47] B. L. Welch. The generalization of "Student's" problem when several different population variances are involved. *Biometrika*, 34(1-2):28–35, 1947.
